# Uneven declines between corals and cryptobenthic fish symbionts from multiple disturbances

**DOI:** 10.1038/s41598-021-95778-x

**Published:** 2021-08-12

**Authors:** Catheline Y. M. Froehlich, O. Selma Klanten, Martin L. Hing, Mark Dowton, Marian Y. L. Wong

**Affiliations:** 1 Faculty of Science, Medicine and Health, University of Wollongong, Wollongong, NSW 2500 Australia; 2 School of Life Sciences, University of Technology Sydney, Sydney, NSW 2007 Australia

**Keywords:** Behavioural ecology, Climate-change ecology, Community ecology, Population dynamics, Behavioural ecology, Climate-change ecology, Community ecology, Population dynamics

## Abstract

With the onset and increasing frequency of multiple disturbances, the recovery potential of critical ecosystem-building species and their mutual symbionts is threatened. Similar effects to both hosts and their symbionts following disturbances have been assumed. However, we report unequal declines between hosts and symbionts throughout multiple climate-driven disturbances in reef-building *Acropora* corals and cryptobenthic coral-dwelling *Gobiodon* gobies. Communities were surveyed before and after consecutive cyclones (2014, 2015) and heatwaves (2016, 2017). After cyclones, coral diameter and goby group size (i.e., the number of gobies within each coral) decreased similarly by 28–30%. After heatwave-induced bleaching, coral diameter decreased substantially (47%) and gobies mostly inhabited corals singly. Despite several coral species persisting after bleaching, all goby species declined, leaving 78% of corals uninhabited. These findings suggest that gobies, which are important mutual symbionts for corals, are unable to cope with consecutive disturbances. This disproportionate decline could lead to ecosystem-level disruptions through loss of key symbiont services to corals.

## Introduction

Multiple disturbances over short periods can disrupt important ecological processes and threaten the persistence of ecosystems^1,2^. From species survival to population bottlenecks and trophic disruptions, such consecutive disturbances may transform entire environments^1–4^. The ability for ecosystems to recover depends on the frequency and intensity of multiple events, which are predicted to increase with climate change^1,5^. Species interactions within complex environments can deteriorate in an accelerated fashion as a result^1^. Whether organisms persist in the short-term during extreme consecutive disturbances will determine their recovery potential and that of associated organisms^6–8^. We need to understand whether ecological relationships are resilient to consecutive disturbances in order to better align future strategies for ecosystem conservation^6,9^.

Mutualism occurs in many taxa and may be one such ecological relationship that proves fragile from consecutive disturbances^6,9^. Mutual symbioses are observed in all environments and promote life in otherwise inhospitable areas^9^. A small shift in environmental conditions may change the nature of such relationships, like mutualism becoming parasitism, or relationships ceasing if one symbiont becomes locally threatened^6^. Climate-driven disturbances can lead to breakdowns of mutualisms like those responsible for preventing seagrass degradation^10^, maintaining myrmecophyte-dominated savannahs^11^, sustaining coral survival^12^, and promoting microbe-assisted biodiversity^9,13^. Collapse of mutual symbioses may have flow-on effects by destabilizing habitats and causing deleterious ecosystem consequences^6,13^. For example, as mutualism breaks down, corals can become more susceptible to stress due to a lack of symbiont services, resulting in fewer corals that provide habitat for other associated species like invertebrates and other fishes, and then these habitats may continue to destabilize as a negative feedback loop exists between reduced coral cover and reduced presence of reef associated species. Studies need to assess the consequences of disturbances on mutual symbioses in order to predict flow-on effects to ecosystems.

Mutually beneficial taxa are especially vulnerable to climate-driven disturbances, but most of the research is primarily focused on an ecosystem’s foundation species instead of associated animals, as seen in coral reefs^2,14–16^. As the frequency and intensity of storms and heatwaves are increasing, corals are being exposed to disturbances in rapid succession^14–16^. Up to 11% of coral reef fishes depend on live corals for survival through food, settlement and shelter^17,18^. In return, coral-associated fishes promote coral resilience by reducing disease, algal growth, and increasing nutrient cycling^19–23^. However, disturbance studies are largely focused on corals^14–16^. If fish symbionts decline disproportionately from climate-driven impacts^24^, then corals will be exposed to additional threats as there is little functional overlap in coral reefs^25^. Disproportional declines in corals and their mutualistic symbionts may lead to ecosystem shifts^26^ if consecutive disruptions become the norm^5^.

**Figure 1:**
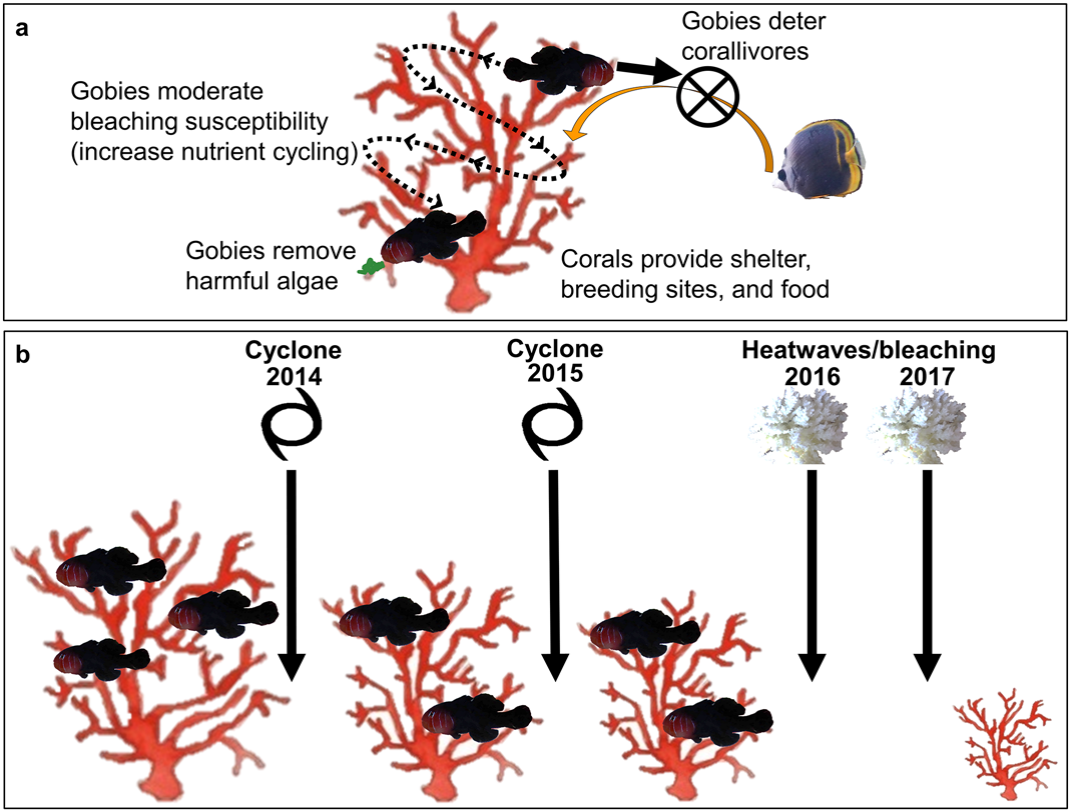
Drastic shifts to the mutual symbiosis of corals and cryptobenthic coral-dwelling gobies following multiple disturbances. (**A**) Benefits that each symbiont receives from the mutual symbiosis^19–21,28,29^. (**B**) Summary of the findings highlighting changes to corals and gobies from each consecutive disturbance with coral-dwelling gobies from the genus *Gobiodon* in scleractinian corals from the genus *Acropora*. Reductions in coral size are drawn to scale and relative to changes in means among disturbances. Figures were illustrated in Microsoft Office PowerPoint 2016.

Here, we examined the impacts of multiple climate-driven disturbances on the persistence of coral-fish symbioses using the most susceptible reef-building corals (genus *Acropora*)^16,27^ and their mutually beneficial inhabitants, cryptobenthic coral-dwelling gobies (genus *Gobiodon*)^20,21^. In return for shelter, breeding sites and food from corals^28,29^, gobies remove harmful seaweed, deter corallivores, and increase nutrient cycling^19–21^ (Fig. 1a). Gobies are often overlooked in disturbance studies because they are small and time-intensive to survey, yet as cryptobenthic fishes they are critical to the trophic structure of coral reefs^4^. We surveyed coral and goby communities throughout four consecutive disturbances at Lizard Island, Great Barrier Reef, Australia. Within four years, the reef experienced two cyclones (2014, 2015), and two unprecedented heatwaves that caused widespread bleaching (2016, 2017)^30^. Our study quantified the additive impacts of cyclones and heatwaves on the persistence of corals and their goby symbionts over a short space of ecological time.

## Results and discussion

### Host and mutual symbionts decline at different rates following consecutive cyclones and bleaching

**Figure 2:**
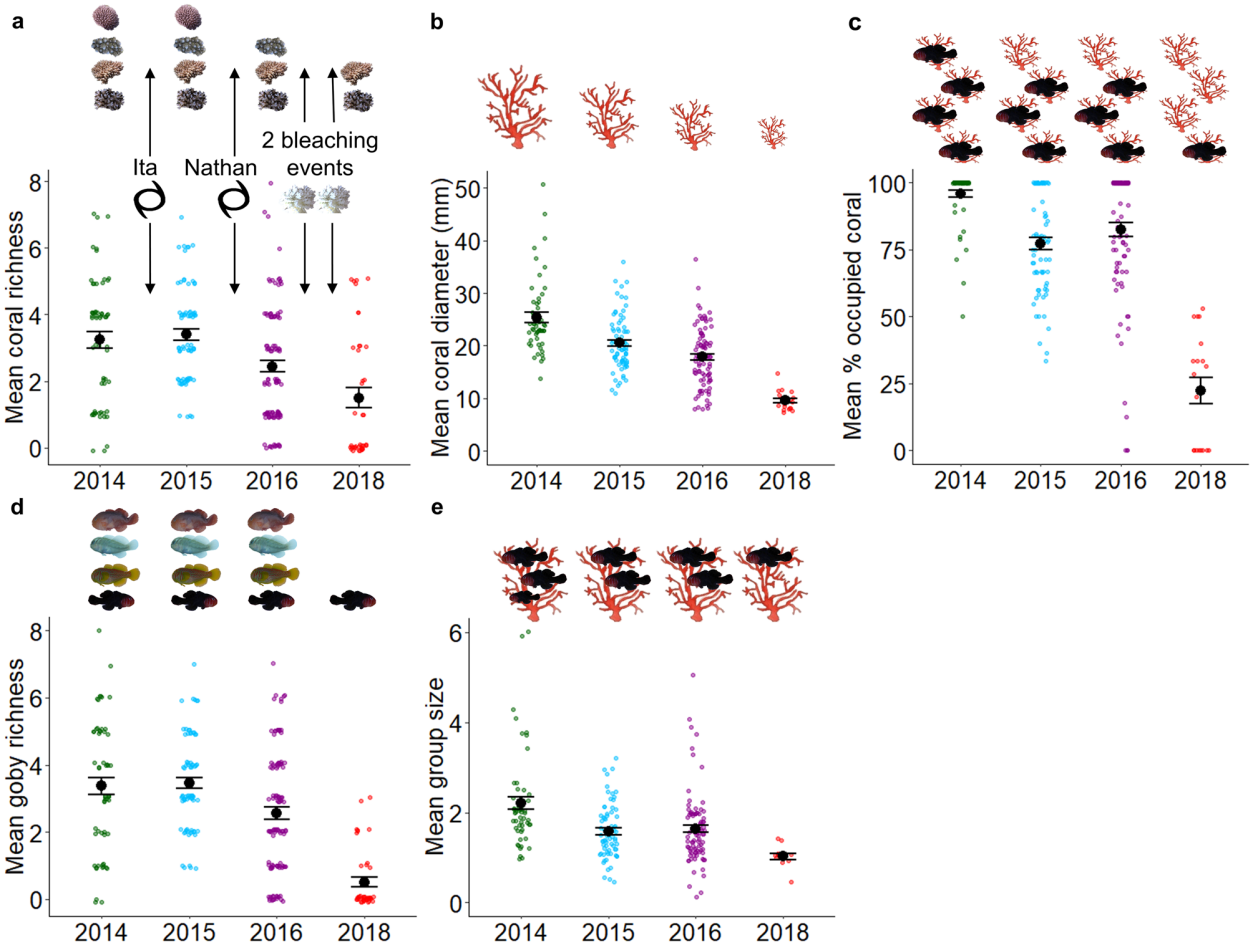
Effects of consecutive climate disturbances on coral and goby populations. Changes in *Acropora* (**a**) richness (n = 279), and (**b**) average diameter (n = 244), (**c**) percent goby occupancy (n = 244) and *Gobiodon* (**d**) richness (n = 279), and (**e**) group size (n = 230) per transect (n = sample size per variable) before and after each cyclone (black cyclone symbols) and after two consecutive heatwaves/bleaching events (white coral symbols) around Lizard Island, Great Barrier Reef, Australia. Error bars are standard error. Fish and coral symbols above each graph illustrate the change in means for each variable among sampling events from post-hoc tests. Figures were illustrated in R (v3.5.2)^33^ and Microsoft Office PowerPoint 2016.

Before and after disturbances, we surveyed *Acropora* corals known to host *Gobiodon* coral gobies along line (30 m) and cross (two 4-m by 1-m belt) transects. In February 2014, prior to cyclones and bleaching events, most of these *Acropora* corals were inhabited by *Gobiodon* coral gobies. Gobies were not found in corals under 7-cm average diameter, therefore we only sampled bigger corals. The vast majority of transects (95%) had *Acropora* corals. On average there were 3.24 ± 0.25 (mean ± standard error) *Acropora* coral species per transect (Fig. 2a) and a total of 17 species were observed among all 2014 transects. Average coral diameter was 25.4 ± 1.0 cm (Fig. 2b), with some corals reaching over 100 cm. Only 4.1 ± 1.4% of corals lacked any goby inhabitants (Fig. 2c). On average there were 3.37 ± 0.26 species of gobies per transect (Fig. 2d) and a total of 13 species among all 2014 transects. In each occupied coral there were 2.20 ± 0.14 gobies (Fig. 2e), with a maximum of 11 individuals of the same species.

In January–February 2015, 9 months after Cyclone Ita (category 4) struck from the north (Supplementary Fig. 1), follow-up surveys revealed no changes to coral richness (p = 0.986, see Supplementary Table 1 for all statistical outputs) relative to February 2014, but corals were 19% smaller (p < 0.001, Fig. 2a,b). Cyclonic activity may have damaged existing corals^31^, which might explain smaller corals. Alternatively, corals may have died from cyclonic damage^31^, but previously undetected corals (less than 7-cm average diameter threshold for surveys) may have grown and accounted for finding smaller corals and no changes to species richness. After the cyclone, gobies occupied 76% of live corals, which meant that occupancy dropped by 19% (p < 0.001, Fig. 2c). Goby richness did not change after the first cyclone relative to February 2014 (p = 0.997, Fig. 2d). However, goby group sizes (i.e., the number of gobies within each coral) were 28% smaller (p < 0.001), with gobies mostly occurring in pairs, and less so in groups (Fig. 2e). Smaller groups were likely due to their coral hosts being smaller than before the cyclones as there is an indirect link between group size and coral size^32^.

In January–February 2016, 10 months after Cyclone Nathan (category 4) struck from the south (Supplementary Fig. 1), our follow-up surveys revealed 26% fewer coral species (p = 0.008), and 13% smaller corals (p = 0.029) relative to February 2015 (Fig. 2a,b). Many corals were damaged (personal observations), and bigger corals were likely heavily damaged and disproportionately reduced in size. As *Acropora* corals vary in several morphological traits such as branch thickness, such characteristics might alter their susceptibility to cyclonic damage^31,34^ and likely explain a decrease in coral richness. There was no change to coral occupancy by gobies relative to February 2015 (p = 0.167, Fig. 2c). Goby richness however did not mirror declines to their coral hosts as there was no change relative to February 2015 (p = 0.060, Fig. 2d). Goby group size did not change relative to February 2015 and most individuals occurred only in pairs (p = 1.000, Fig. 2e). Since the second cyclone did not add additional changes to coral occupancy, goby richness or goby group size, gobies may have exhibited some ecological memory^30^ from the first cyclone. However, when combining the effects of consecutive cyclones, coral and goby symbioses were disrupted substantially. Coral hosts were 30% smaller relative to 2014 (pre-disturbances), 25% of hosts were uninhabited compared to only 4% in 2014, and goby group size remained the same as after the first cyclone whereby gobies were no longer living in groups, instead living in pairs (Fig. 1b). These acute disturbances had effects lasting longer than 10 months and will likely require many years to return to pre-disturbance status^14^.

Unfortunately, there was no time for recovery from cyclones before two prolonged heatwaves caused widespread bleaching in March–April 2016 and February–May 2017 (Supplementary Fig. 1). Ten months after the second bleaching event (Jan–Feb 2018), we returned to Lizard Island and rarely found live corals along our transects. Half (50%) of the transects lacked any living *Acropora* corals compared to just 5% of transects before any disturbance (2014). There were 39% fewer coral species (p = 0.009) relative to February 2016, with only 1.5 ± 0.31 species per transect (Fig. 2a). Corals were 47% smaller than in February 2016 (p < 0.001, Figs. 1b, 2b), averaging 9.57 ± 0.39 cm coral diameter (maximum 21 cm). Acroporids were also the most susceptible family to bleaching from these back-to-back heatwaves across the Great Barrier Reef and their coral recruitment was at an all-time low^2,16^. Since corals were lethally bleached during the prolonged heat stress, only a few acroporids species survived these consecutive events^35^. Such declines and extensive bleaching from the 2015–2016 heatwave were also observed in many areas around the world^5,36^.

After consecutive heatwaves, coral gobies faced even more drastic declines than their coral hosts in all our survey variables. Of the few live corals recorded, most (77.7 ± 4.8%) corals lacked gobies compared to just 4% without gobies pre-disturbance (2014), and 24% after cyclones (p < 0.001, Fig. 2c). For the first time, only after heatwaves, we observed a change in goby richness with 80% fewer goby species per transects relative to February 2016 (p < 0.001, Fig. 2d), even though consecutive cyclones did not affect goby richness. Alarmingly, goby group size decreased to such an extent that gobies were no longer found in groups (p = 0.036), rarely in pairs (n = 3), and the few observed occurred singly (Fig. 2e). For these long-living, monogamous, and nest brooding fishes^28,37^, finding gobies predominantly without mates suggests that reproduction likely ceased or was significantly delayed for most individuals in the population^28^. An interruption in mate pairing likely led to extremely low recruitment and turnover rates in gobies from climatic disturbances.

Gobies declined substantially more than coral hosts after consecutive heatwaves, leaving most corals uninhabited (Fig. 1b). Although communities still had not recovered from cyclonic disturbances before prolonged heatwaves, we suspect that heatwaves had more devastating impacts on gobies than cyclones. Gobies have a strong tendency to stay in the same coral they settle in as recruits^38^ as long as the coral is alive^39^, yet many may have unsuccessfully attempted to find other corals once their coral was lethally bleached^4^. Unlike gobies, other coral-dwelling fishes, like damselfish recruits, successfully adopted alternative habitat, including dead corals^40^. Gobies did not adopt alternative habitat and were surprisingly absent from most living corals.

Importantly, goby richness did not change after consecutive cyclones and only changed after heatwaves. Thus coral host death likely is not the only stressor and gobies may have suffered physiological consequences from prolonged environmental disturbances^41–43^. Although gobies can survive short exposures of hypoxia^44^, extended periods of reduced wind-induced mixing and thermal stress may jeopardize physiological functioning^45,46^. Indeed, reef fishes can lose the ability to detect predators, kin, and habitat^41–43^, and to reproduce from environmental stress^46^. Gobies likely lost similar functioning from heatwaves leading to high mortality and little goby turnover, which left many healthy corals unoccupied. A lack of mutual goby symbionts following consecutive disturbances suggests that coral hosts may begin experiencing additional threats to their recovery^19–21^. Such declines and potential physiological consequences may also hold true for other coral-dwelling organisms, like symbiotic xanthid crabs^47^. Since acroporid corals are crucial foundation species for coral reef ecosystems, greater declines in their symbionts from multiple disturbances may reduce the persistence of corals and destabilize habitats over large scales.

### Communities of goby symbionts exhibit greater changes than communities of coral hosts from multiple disturbances

**Figure 3:**
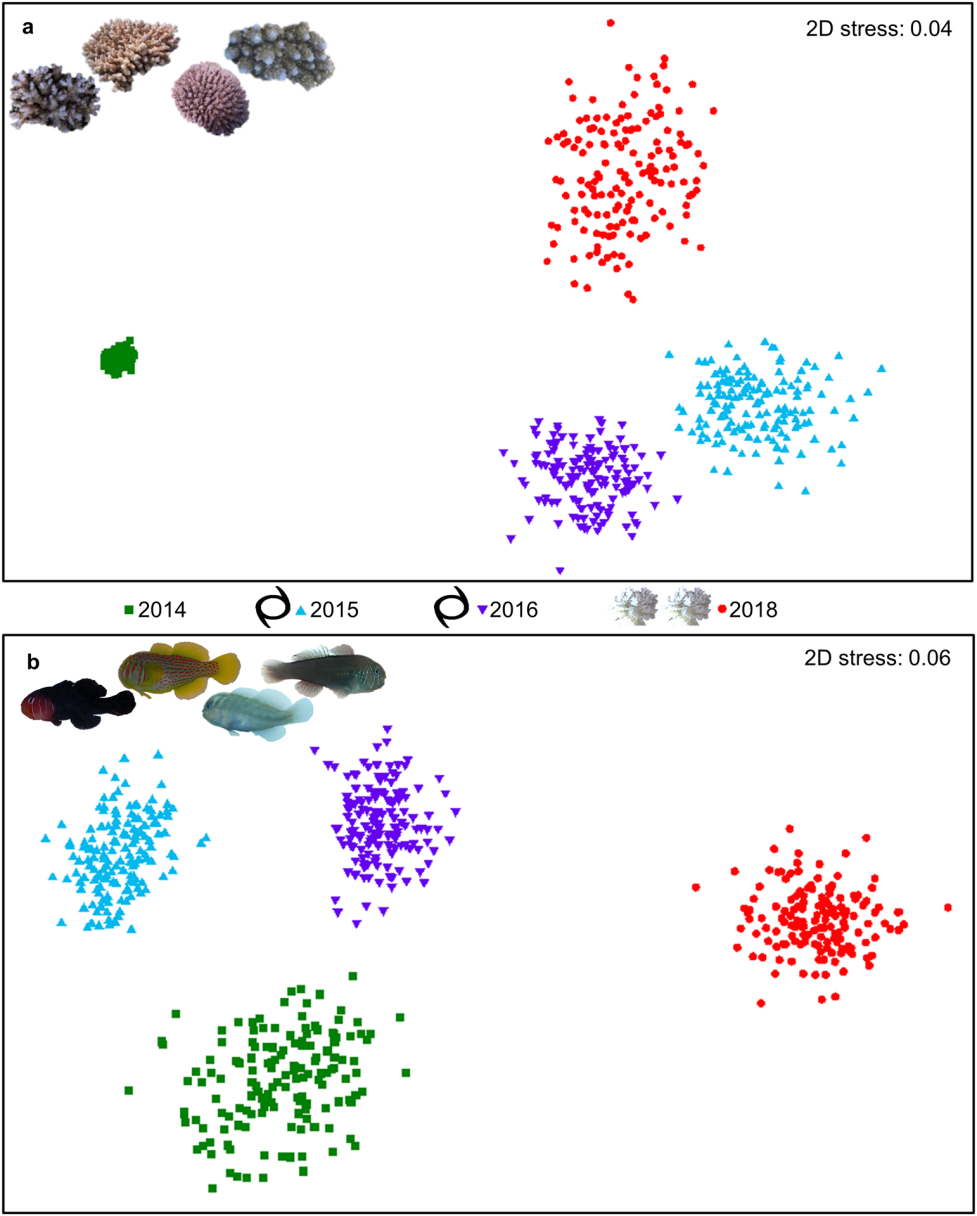
Shifts in communities of corals and gobies throughout consecutive climate disturbances. The changes in communities along transects (n = 279) before and after each cyclone (black cyclone symbols) and after two consecutive heatwaves/bleaching events (white coral symbols) at Lizard Island, Great Barrier Reef, Australia, for (**a**) *Acropora* corals and (**b**) *Gobiodon* gobies visualized on non-metric multidimensional scaling plots. Each colored point represents a single transect, points represent bootstrapped data, and points closer together are more similar in species composition than points further apart. Figures were illustrated in PRIMER-E software (v7, https://www.primer-e.com/) and Microsoft Office PowerPoint 2016.

In February 2014, before the consecutive climatic events, we recorded 17 species of *Acropora* corals known to host *Gobiodon* coral gobies, with the most common being *A. gemmifera, A. valida, A. millepora, A. loripes, A. nasuta, A. intermedia, A. tenuis,* and *A. cerealis*. Thirteen species of *Gobiodon* gobies were recorded, with the most common being *G. rivulatus, G. fuscoruber, G. brochus, G. histrio, G. quinquestrigatus,* and *G. erythrospilus*. Each disturbance changed the assemblages of both corals (p < 0.001, Fig. 3a) and gobies (p < 0.001, Fig. 3b), yet the changes in both corals and gobies did not mirror each other since communities among sampling events did not aggregate similarly (Fig. 3).

**Figure 4:**
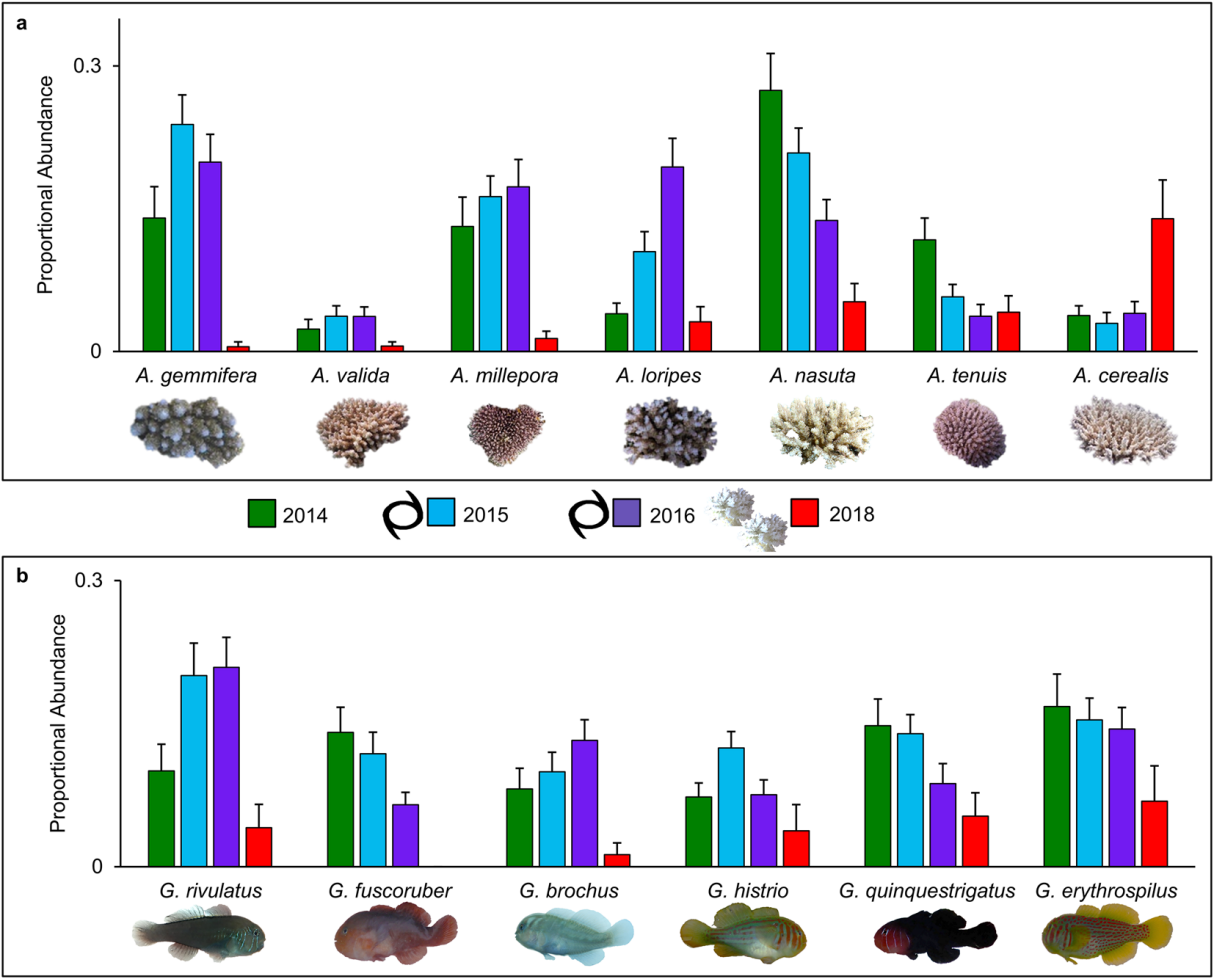
Changes in abundance of coral and goby species before and after each consecutive climate disturbances. The proportional abundances for the most common species within each transect (n = 279) for (**a**) *Acropora* corals and (**b**) *Gobiodon* gobies is shown before and after each disturbance around Lizard Island, Great Barrier Reef, Australia: effect of each cyclone (black cyclone symbols) and combined effect of two consecutive heatwaves/bleaching events (white coral symbols). Proportional abundances were calculated by taking the count per species per transect divided by the total count among all species observed per transect. Visualized here are the proportional abundances pooled per sampling event for the most common species. Figures were illustrated in Microsoft Office PowerPoint 2016.

After the first cyclone, 11 *Acropora* species were found, and the common species increased in proportional abundance relative to February 2014 (p = 0.009, Figs. 3a, 4a). The previously rare species *A. valida* increased in proportional abundance as well. However, *Acropora intermedia*, which was previously recorded in several transects, was no longer observed; this is likely due to its branches being long and thin, thus highly susceptibility to damage^31^. Goby assemblages were also altered after the first cyclone (p = 0.003, Fig. 3b), and the proportional abundance of the common species differed in response relative to 2014 (Fig. 4b). The proportion of *G. histrio* and *G. rivulatus* in transects increased compared to 2014, and so did the proportion of their preferred hosts, *A. nasuta* and *A. gemmifera*, respectively (Fig. 4)^48^. However, the proportion of *G. fuscoruber* decreased even though its common host, *A. millepora*^48^, was recorded more frequently than several other corals (Fig. 4). *Gobiodon fuscoruber* is a group-living species, and it is possible that as group size decreased, they were outcompeted for coral hosts by other species^49^. Two rare gobies were no longer recorded (*G. citrinus* and *G. okinawae*), and both preferred *A. intermedia*^48^, which also disappeared. Since species of both corals and gobies had mixed responses to the cyclone, there may be some positive effects of an intermediate level of disturbance for those species that increased in proportional abundance, specific to the intermediate disturbance hypothesis^50^.

After the second cyclone, we found mixed results in coral assemblages (p < 0.001, Fig. 3a). Although 15 *Acropora* species were found after the second cyclone (5 more than after the previous cyclone) and no species were locally extirpated, only *A. loripes* became more common (Fig. 4a). Several of the most common corals (i.e. *A. gemmifera*,* A. nasuta*,* A. tenuis*) decreased in proportional abundance after the second cyclone (Fig. 4a). Goby communities were altered once again (p < 0.001, Fig. 3b), this time with fewer species increasing in proportional abundance and more species decreasing (Fig. 4b). However, all *Gobiodon* species were encountered, even *G. citrinus* and *G. okinawae* that originally disappeared after the first cyclone. *Gobiodon brochus* increased in proportional abundance and so did its common host *A. loripes*^48^. However, *G. rivulatus* increased even though its preferred host *A. gemmifera* decreased (Fig. 4)^48^.

After consecutive bleaching events, the reef was left with few corals, most of which were very small in size. Although the coral community after bleaching was distinct from each disturbance sampling event (p < 0.001), all disturbed communities aggregated closely together compared to the pre-disturbance community (2014, Fig. 3a). After bleaching, the most coral species were recorded (22 in total) compared to all other sampling events. Although coral richness per transect was the lowest after bleaching (Fig. 2a), the coral community as a whole was more diverse and was made up of more coral species. A few *A. intermedia* were again recorded after none were observed following the first cyclone, along with 9 rare and previously unrecorded *Acropora* species. However, some species were no longer observed, e.g. *A. divaricata* (previously rare)*, A. granulosa* (previously rare)*,* and *A. humilis* (previously common). Many of the common coral species became rare after bleaching (Fig. 4a). In coral reefs, *Acropora* are one of the most susceptible coral genera to cyclone damage and bleaching in a warming climate^16,31^, which explains such steep declines in many *Acropora* species. Surprisingly, *A. cerealis*, which was previously rare, had since increased in proportional abundance despite multiple disturbances (Fig. 4a). In other areas though, such as the Andaman Bay, *A. cerealis* was one of the most lethally bleached species^36^. Regional differences in thermal plasticity and coral recruitment may have disproportionately affected the survival thresholds of identical species.

Coral gobies were more dramatically affected by consecutive bleaching than corals. Goby communities after bleaching were the most distinct (p < 0.001), while communities from all other sampling events aggregated closer together (Fig. 3b). Every goby species declined after bleaching (Fig. 4b), and half of the species were no longer recorded. Some species were locally extirpated, including *G. citrinus* (previously rare), *G. sp. D* (previously rare), *G. bilineatus* (previously common), and *G. fuscoruber* (previously common, Fig. 4b). None of the locally extirpated species were observed during random searches. Only 6 species remained, and no previously unrecorded species were observed. As expected, gobies were never found in dead corals, as they can only survive in live corals (albeit surviving in stressed corals^39^). These findings highlight the greater impact that multiple disturbances have on symbiont communities, especially when disturbances are a mix of acute (short-term) and prolonged (long-term) events. Although we cannot assess the effects of cyclones compared to heatwaves since they occurred in succession, we can clearly show that multiple disturbances affect corals and gobies differently. We observed a loss of biodiversity for gobies from multiple disturbances, whereas their coral hosts were more diverse even though fewer corals were recorded and they were smaller.

The study demonstrates the effects that multiple disturbances have on reef ecosystems down to the level of important mutualisms. Disturbance studies have primarily focused on the disturbance effects to corals^16,30,31^, yet cryptobenthic fishes are often overlooked^4^. We may be missing effects of disturbances on fishes that could have flow-on effects on the whole ecosystem, especially since cryptic fishes make up a large portion of reef biodiversity and are crucial prey for many taxa^4^. This study is one of few multi-year studies to record species-level changes in cryptobenthic fishes from multiple consecutive disturbances. Intriguingly, although corals and gobies responded similarly at first to the initial two cyclones, they then diverged in their responses after additional stress from heatwaves. Here we show that gobies declined faster on a community and species level than their coral hosts, which will likely leave corals exposed to algal growth, poor nutrient cycling, and corallivory^19–21^ (Fig. 1). The unwillingness of gobies to use alternative habitat in the short-term may drastically reduce their resilience to disturbances, threatening localized extinction^51^. Declines from a single disturbance have the potential for a resilience, but multiple events will require long-term recovery^31,32^ as most corals are uninhabited after consecutive disturbances (Fig. 1b). Although the disturbances in this study were compounded, heatwaves may have had an even stronger effect on gobies since goby communities differed the most after the heatwaves, whereas coral communities remained similarly diverse after each disturbance. Without the added benefits of gobies, surviving corals will likely experience further threats to survival^19–21^. Multiple disturbances may even cause ecosystem shifts when the foundation species of the environment, such as hard corals, face extreme declines^6^. If mutual symbionts show greater declines than corals as seen in this study, important processes may be exacerbated, further jeopardizing the recovery potential of an ecosystem’s foundation species.

### Future implications for symbiotic relationships from multiple disturbances

Our study demonstrates that consecutive disturbances result in uneven declines between mutual symbionts, and this has the potential for exposing surviving hosts to additional threats if their mutual and cryptic inhabitants disappear. As mutualisms break down, organisms that rely on these mutualisms may become more vulnerable to multiple disturbances and there may be ecosystem-level disruptions as a result^1,6,13,24^, especially as climate-driven events becomes more frequent^5^. Although the length and type of the disturbance play important roles in disturbance impacts, few studies have examined the effect of multiple disturbances^30,31,52^. If successive threats become the norm, a system will already be stressed before a second event strikes, leading to greater consequences^31^. Population bottlenecks will inevitably follow^3^ and threaten the survival of many organisms globally^7^. Flow-on effects will affect closely-associated organisms, especially for those that depend on feedback loops with symbionts^6^. In each ecosystem, species are responding differently to disturbances, and mutually beneficial relationships are being tested^6^. Our study suggests that multiple disturbances will likely leave ecosystem builders exposed to additional threats if their cryptic symbionts fail to recover.

## Methods

### Study location and sampling effort

The study was completed at Lizard Island, Great Barrier Reef, Queensland, Australia (14° 40.729′ S, 145° 26.907′ E, Supplementary Fig. 1). Four climatic events affected Lizard Island from 2014 to 2017. Cyclone Ita hit in April 2014, and Cyclone Nathan hit in March 2015 (Supplementary Fig. 1). The following year, the first extensive mass-bleaching event spanned March to April 2016, and a second extensive mass-bleaching event spanned February to May 2017. A total of 17 sites were first visited in February 2014 before climatic events. After the first cyclone 10 sites were revisited in January–February 2015, 15 sites in January–February 2016 (after second cyclone), and 17 sites in February–March 2018 (after back-to-back heatwaves).

### Survey method

At each site, goby and coral communities were surveyed visually within 1 m on either side of 30-m line transects by two experienced scuba divers in 2014 (n = 59 transects) and were repeated in 2018 (n = 40). Transects were completed in 2015 (n = 73) and 2016 (n = 107) using a different method: cross-transects—two 4-m × 1-m belt transects laid in a cross around a focal colony. Not all sites were surveyed during each sampling event due to weather conditions and cyclones scouring sections of reef down to bare rock after their impacts. Transects at all sites were completed on the reef flat, crest, and slope and were within 1 to 6-m in depth. In 2018, random searching for up to one hour (in addition to the transects) was also completed in several areas (n = 28 searches) to determine whether goby species that were missing were simply absent from transects or were instead likely locally extirpated from Lizard Island. For all methods, when a live *Acropora* coral was encountered, the coral was identified to species and measured along three dimensions: width, length, and height^29^. A bright torch light (Bigblue AL1200NP) was shone in the coral to quantify the number of goby residents and the *Gobiodon* species inhabiting each coral. Gobies were delineated either as adults or recruits depending on their coloration and size. The study was performed in accordance with relevant guidelines and regulations, including ARRIVE guidelines, under the University of Wollongong Animal Ethics protocol AE1404 and AE 1725 and under research permits issued by the Great Barrier Reef Marine Park Authority (G13/36197.1 and G15/37533.1).

### Data analysis

Univariate analyses were completed to assess changes in the following variables per transect (as a single sample) throughout disturbances: adult goby species richness, average adult goby group size per coral, percent occupied coral, coral species richness, average coral diameter (the three coral dimensional measurements were averaged to calculate an average diameter^29^). Goby and coral richness were count data with several zero data points after multiple disturbances. As such, richness variables were each analyzed using zero-inflated generalized linear mixed model designs (GLMER: using poisson family) among sampling year (fixed factor) and site (random factor). The following variables were continuous variables and as such were analyzed using linear mixed model designs (LMER) amongst the sampling year (fixed) and site (random): average coral diameter, average goby group size, and percent occupied corals. Variables analyzed with LMER were transformed as required to meet normality and homoscedasticity, which were determined using Q-Q plots, histograms, and residuals over fitted plots. Tukey’s tests were used for differentiating between statistically significant levels within factors. For each univariate analysis, outliers were investigated if their standard residuals fell outside of 2.5 standard deviation from 0 and were subsequently removed. A maximum of 7 outliers were removed for any given analysis. All analyses were completed in R (v3.5.2)^33^ with the following packages: tidyverse^53^, lme4^54^, lmerTest^55^, LMERConvenienceFunctions^56^, piecewiseSEM^57^, glmmTMB^58^, emmeans^59^, DHARMa^60^, and performance^61^.

Community composition was analyzed separately for corals and gobies. To take into account the different survey techniques, samples were standardized to create proportional abundance as follows: for each survey, we divided each count per species by the total abundance of all species. Only adult gobies were included in the analyses. Communities were analyzed with permutational analyses of variance (PERMANOVA). Communities were compared against sampling year (fixed factor) and were controlled for site (random factor) with permutational analyses of variance in PRIMER-E software (v7). Type I error was included because of the unbalanced design with uneven transects per year. Community differences were bootstrapped to a 95% region for a total of 150 bootstraps per year and were visualized on non-metric multidimensional scaling plots. When statistical differences were observed, similarity percentage analyses (SIMPER) were performed to determine what species contributed to the differences observed. Species contributions were cut off to the top 75% of species that contributed the most to differences observed. See Supplemental Table 1 for all statistical outputs of univariate and multivariate analyses.

## Supplementary Information

Supplementary Information.

## Data Availability

Data and statistical coding are available at the Knowledge Network for Biocomplexity repository with identifier: https://doi.org/10.5063/1R6NZ1.
