# Supplementary material for: Uneven declines between corals and cryptobenthic fish symbionts from multiple disturbances
Source: Sci Rep. 2021 Aug 12;11:16420. doi: 10.1038/s41598-021-95778-x (PMC8361158; doi:10.1038/s41598-021-95778-x)
Supplement: Supplementary file 1 — Supplementary Information. [file 41598_2021_95778_MOESM1_ESM.pdf]

<u>SUPPLEMENTARY MATERIAL FOR ARTICLE:</u>

Uneven declines between corals and cryptobenthic fish symbionts from multiple disturbances

Authored by: Catheline Y.M. Froehlich*, O. Selma Klanten, Martin L. Hing, Mark Dowton, and Marian Y.L. Wong

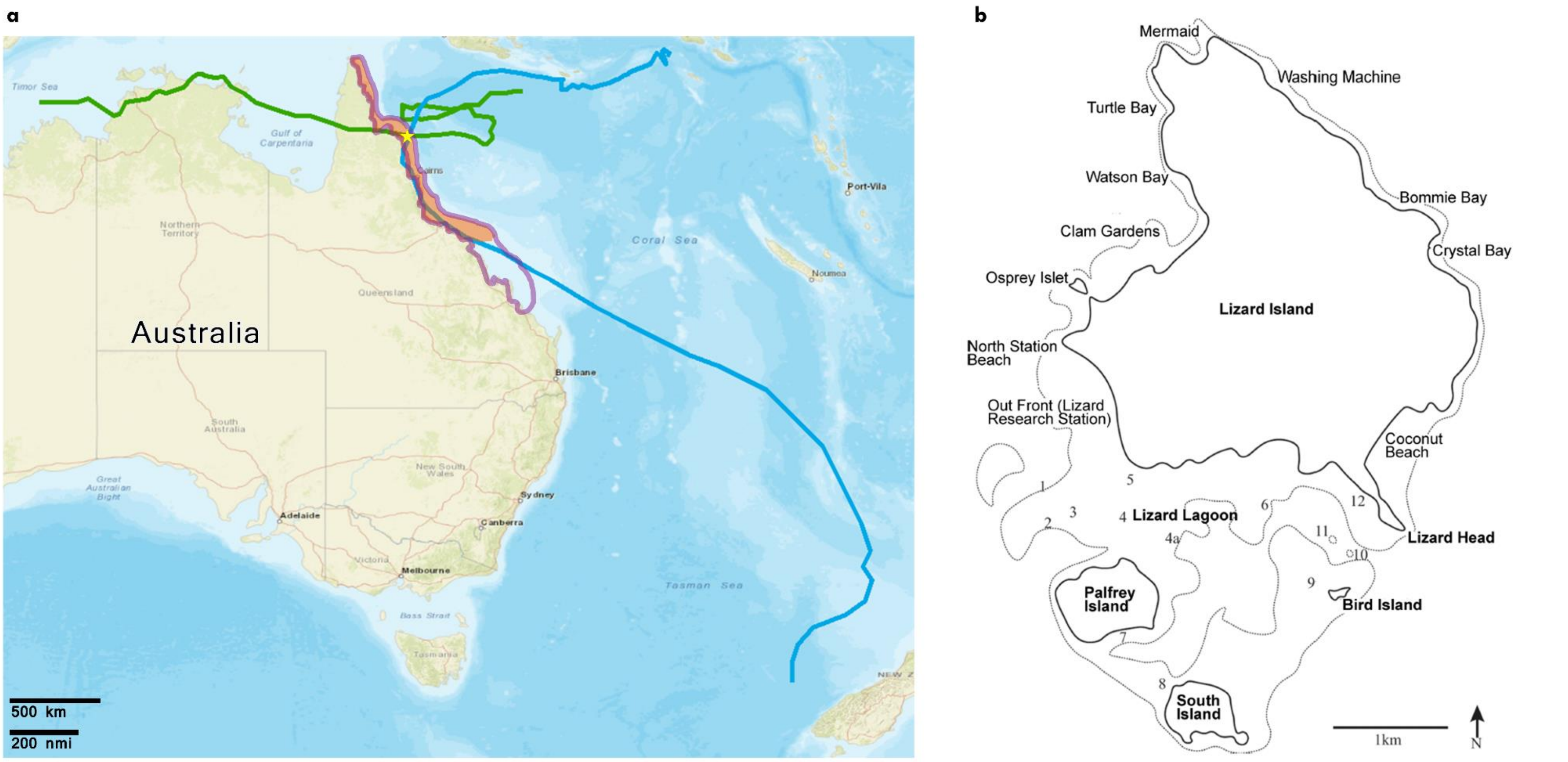

Supplementary Figure 1. Map and location of consecutive climate disturbances affecting Lizard Island, QLD, Australia. a Map of Australia created in ArcGIS (source: Esri, DeLorme, HERE, Garmin, USGS, Intermap, iPC, NRCAN, Esri Japan, METI, Esri China (Hong Kong), Esri (Korea), Esri (Thailand), MapmyIndia, Tomtom, NGCC, OpenStreetMap contributors, and the GIS User Community) with cyclone tracks of Ita 2014 (blue line) and Nathan 2015 (green line) sourced from the Australian Government Bureau of Meteorology (http://www.bom.gov.au/cyclone/history/tracks/beta/?region=sh), with areas overlayed to show impacts of coral bleaching from 2016 (orange shaded area) and 2017 (purple outlined area). Lizard Island is highlighted with yellow star. Bleaching areas were adapted from two papers[15,16]. **b** Map of Lizard Island (-14.687264, 145.447039) created in Adobe Illustrator® (v2020). Numbers represent discrete sites as follows: Big Vickey's Reef (1); Vickey's Reef (2); Horseshoe Reef (3); Palfrey Reef (4±4a); Loomis Reef (5); Trawler (6); Picnic Beach (7); Ghost Beach (8); Bird Island Reef (9); Entrance Bommie (10); Bird Bommie (11); Lizard Head (12).

Supplementary Table 1. Statistical output of all univariate and multivariate analyses for changes in *Acropora* corals and coral-dwelling *Gobiodon* gobies following multiple disturbances. Corals and gobies were surveyed before (Feb 2014) and after each cyclone (Jan-Feb 2015, Jan-Feb 2016) and consecutive bleaching (Feb-Mar 2018) around Lizard Island, Great Barrier Reef, Australia. CI means confidence interval.

Response Variable	Model	Predictor variable	Factor Type	df	Model Statistic	Test-value	p-value	R-squared	Pairwise Comparison	Pairwise Statistic	Test-value	P-value	Lower 95% CI	Upper 95% CI
Coral Richness	GLMM	Year	fixed	3	χ^2 (chi-	35.418	< 0.0001	0.147	2014, 2015	t-ratio	-0.34	0.9864	-0.307	0.236
	poisson	Site	random		squared)			marginal	2014, 2016		2.658	0.0412	0.007	0.53
									2014, 2018		4.875	0.0001	0.349	1.137
				Sample	size	n = 279			2015, 2016		3.203	0.0083	0.059	0.55
				Outliers	removed	n = 5			2015, 2018		5.09	<.0001	0.383	1.174
									2016, 2018		3.18	0.0089	0.089	0.86
Average Coral	LMM	Year	fixed	3	F-value	67.207	< 0.0001	0.5874422	2014, 2015	t-ratio	6.6	<.0001	0.19419	0.445
Diameter		Site	random					conditional	2014, 2016		9.572	<.0001	0.31755	0.553
log-transformed									2014, 2018		13.334	<.0001	0.72185	1.07
				Sample	size	n = 244			2015, 2016		2.797	0.0285	0.00863	0.223
				Outliers	removed	n = 7			2015, 2018		8.406	<.0001	0.39888	0.754
									2016, 2018		7.086	<.0001	0.2923	0.629
Coral Assemblage	PERMANOVA	Year	fixed	(3,259)	pseudo-F	10.361	0.0001	N/A	2014, 2015	t-value	5.8509	0.0001	N/A	N/A
		Site	Random	(16,259)		2.7032	0.0001		2014, 2016		4.0838	0.0001		
									2014, 2018		4.6241	0.0001		
				Sample	size	n = 279			2015, 2016		1.7032	0.0092		
									2015, 2018		1.704	0.0066		
									2016, 2018		2.1477	0.0002		
Goby Richness	GLMM	Year	fixed	3	χ^2 (chi-	99.332	< 0.0001	0.524	2014, 2015	t-ratio	0.195	0.9974	-0.249	0.289
	poisson	Site	random		squared)			marginal	2014, 2016		2.545	0.0555	-0.004	0.512
									2014, 2018		7.999	<.0001	1.277	2.496
				Sample	size	n = 279			2015, 2016		2.512	0.0602	-0.007	0.475
				Outliers	removed	n = 3			2015, 2018		7.886	<.0001	1.254	2.478
									2016, 2018		6.975	<.0001	1.027	2.237
Average Goby	LMM	Year	fixed	3	F-value	12,877	< 0.0001	0.2007437	2014, 2015	t-ratio	4.308	0.0001	0.1420	0.569
Group Size		Site	random					conditional	2014, 2016		4.645	<.0001	0.1603	0.564
log-transformed									2014, 2018		5.088	<.0001	0.3688	1.133
				Sample	size	n = 230			2015, 2016		0.088	0.9998	-0.1802	0.193
				Outliers	removed	n = 3			2015, 2018		2.683	0.0389	0.0140	0.776
									2016, 2018		2.709	0.0364	0.0172	0.760
Percent Occupied	LMM	Year	fixed	3	F-value	79.009	< 0.0001	0.6253415	2014, 2015	t-ratio	5.876	<.0001	10.88	28
Coral		Site	random					conditional	2014, 2016		4.394	<.0001	5.55	21.5
									2014, 2018		15.143	<.0001	59.63	84.2
				Sample	size	n = 244			2015, 2016		-2.066	0.1674	-13.37	1.5
				Outliers	removed	n = 6			2015, 2018		10.739	<.0001	39.84	65.1
									2016, 2018		12.625	<.0001	46.45	70.4
Goby Assemblage	PERMANOVA	Year	fixed	(3,259)	pseudo-F	5.8348	0.0001	N/A	2014, 2015	t-value	1.709	0.0061	N/A	N/A
		Site	Random	(16,259)		2.2043	0.0001		2014, 2016		1.777	0.0042		
									2014, 2018		2.9856	0.0001		
				Sample	size	n = 279			2015, 2016		2.1039	0.0008		
									2015, 2018		3.6387	0.0001		
									2016, 2018		2.7908	0.0001		
